# Developing a short form of the Awe Experience Scale (AWE-SF) in psychedelic samples

**DOI:** 10.1371/journal.pone.0314469

**Published:** 2024-12-04

**Authors:** Marianna Graziosi, Julia Sarah Rohde, Stephanie Lake, Philippe Lucas, Scott Barry Kaufman, David Bryce Yaden

**Affiliations:** 1 Center for Psychedelic and Consciousness Research, Department of Psychiatry and Behavioral Sciences, Johns Hopkins School of Medicine, Baltimore, MD, United States of America; 2 Department of Clinical Psychology, Hofstra University, Hempstead, NY, United States of America; 3 Department of Clinical Psychology, Virginia Tech University, Blacksburg, VA, United States of America; 4 UCLA Center for Cannabis and Cannabinoids, Jane and Terry Semel Institute for Neuroscience and Human Behavior, Los Angeles, CA, United States of America; 5 Department of Psychiatry and Biobehavioral Sciences, David Geffen School of Medicine, UCLA, Los Angeles, CA, United States of America; 6 Michigan Psychedelic Center, University of Michigan, Ann Arbor, MI, United States of America; 7 Columbia University, New York, NY, United States of America; Polytechnique Montreal, CANADA

## Abstract

This study aimed to develop and validate a short-form version of the AWE-S (AWE-SF) within psychedelic samples, to reduce participant burden while maintaining psychometric integrity. Across five studies, we first replicated the original six-factor structure of the AWE-S through exploratory and confirmatory factor analyses (Study 1), leading to the creation of the 12-item AWE-SF (Study 2–3). We then established the AWE-SF’s initial and predictive validity by correlating it with relevant emotional, psychedelic, and well-being outcomes (Study 4–6). The AWE-SF demonstrated strong positive associations with positive emotions and was also linked to openness to experience. Moreover, the AWE-SF effectively predicted both mystical-type and challenging psychedelic experiences, as well as long-term well-being outcomes such as life satisfaction and psychological richness. In particular, the facets of connection and vastness were associated with positive emotional states and mystical-type experience, while accommodation and self-loss were associated with negative emotional states and challenging psychedelic experience. These findings suggest that the AWE-SF is a robust, reliable, and accessible tool for measuring awe experience.

## Introduction

The landmark conceptual article on awe published in 2003 by Keltner and Haidt [1] was the point of entry into the modern empirical literature for this complex emotion as a psychological construct. In their article, they describe awe as a cognitive-emotional process, which involves: 1) an encounter with some sort of perceptually vast stimulus (i.e., *vastness)*; and 2) a need for that stimulus to be accommodated (i.e., *accommodation* [2]. In other words, according to the modern psychological understanding of awe, it is an experience evoked by a stimulus that is so vast that it urges us to alter our very understanding of the world and/or our place in it. Furthermore, awe is a “complex” or “mixed” emotion because it involves positive and negative features [2]. In addition to positive forms of wonder and amazement typical of awe experience, researchers have demonstrated a threat-based, negative variant of awe, which is elicited by stimuli such as tornadoes and natural disasters [3] and that individuals may experience both fear and awe simultaneously [4].

There are other facets of awe beyond the two necessary, definitional features (i.e., *vastness* and *accommodation*). Yaden and colleagues [5] identified additional qualities of awe from the empirical literature (i.e., *self-diminishment*, *connectedness*, *altered time perception*, and *physiological changes*) and investigated through factor analysis, including them (along with vastness and accommodation) in the Awe Experience Scales (AWE-S). Subsequent work on awe has further described the various facets of awe in a variety of contexts. Each of these facets of awe are described in what follows.

### Vastness

In the definition outlined by Keltner and Haidt [1] one necessary condition of awe is that of vastness. Research has shown that awe can be elicited by *literally vast* objects, such as nature [6, 7] or space [8, 9] as well as *figuratively vast* objects, such as spiritual [10, 11] or psychedelic [12, 13] experience or grand theories [2], and finally *relatively vast* objects such as interpersonally significant experiences with loved ones (e.g., seeing your child walk for the first time [12].

### Accommodation

The second necessary condition of awe (i.e., accommodation) is that it necessitates that one alters some aspect of their mental representation of the world. Research has also suggested that awe relates to learning [14, 15], which would also support this link. Another study found that compared with other positive emotions (e.g., amusement, contentment, and pride), awe challenged or changed the way that participants viewed the world (e.g., experiencing something that they did not believe was possible [14] or readjusting one’s habitual sense of scale. Finally, recent work by Danvers and Shiota [16] demonstrates that awe decreases the likelihood that participants would rely on internal knowledge (i.e., knowledge that they already had before an experience) when processing new events. This means that individuals experiencing awe are *less* likely to use past learning when trying to understand and make sense of new experiences.

### Small self

That awe induction leads to a small sense of self (i.e., self-diminishment or self-loss or reduced self-salience) has been empirically demonstrated in a wide variety of studies [11, 17–19]. More importantly, scores on this small sense of self were found to be significantly higher for awe when compared to the other positive emotions of amusement, contentment, gratitude, interest, joy, love, and pride [14].

### Time perception

Awe has also been found to alter perceptions in various other ways. First, awe has been related to an altered perception of time, where participants feel time as taking on an expansiveness, dilating [6], or slowing down [17]. van Elk and Rotteveel [20] have called into question the degree to which “lab-induced” awe affects perceived time dilation. These authors found mixed results, where stronger ratings of awe were associated in perceived time dilation in one study, however, this finding was not replicated in a second study. However, a recent study by Gill and colleagues [21] demonstrated that individuals perceived time to last *longer* in duration (via participant estimates of time) when they experienced images of vast landscapes, such as the Pacific Ocean or the Grand Canyon. Across three studies these authors demonstrated that awe experience mediated the relationship between vastness and perceived time.

### Physiological changes

A subset of physiological reactions have come to signify awe experience, such as: facial expressions including raised inner eyebrows, widened eyes, a slightly open jaw, a visible inhalation, and a leaning forward with the head [22]; goosebumps [23]; changes in autonomic nervous system responding [24]; and even perception of the body as smaller [25] and as being slower or immobile [17].

### Connectedness

Awe is not only considered a complex emotion, but it also sits on a continuum of experiences described as self-transcendent. This so-called ‘unitary continuum,’ which includes constructs with many differences but which are similar insofar as they involve reduced self-salience and increased connectedness, proceeds from less intense self-transcendent experiences such as mindfulness, and flow states to peak experiences, and mystical-type experiences [26]. The authors situate awe somewhere in the middle and describe awe as a self-transcendent positive emotion that has “a particularly intense self-transcendent quality” (p. 5). Awe also has been found to increase feelings of connectedness, such as increases in collective engagement and feelings of common humanity [18, 27].

## Outcomes related to awe

There are numerous outcomes that have been reported to arise out of the experience of awe, such as improvements in mood, well-being, life-satisfaction. One study found that awe-induction increased perceived time-availability, reduced impatience and led to increases in perceived life-satisfaction [6]. In recent work by Anderson, Monroy, and Keltner [27] awe experienced by military veterans and underserved youth predicted these participants’ increased well-being scores and decreased stress responses at a one-week follow-up. Other work has found that awe-induction led to positive effects on mood, as well as serving as a buffer against negative mood [28, 29], and has been shown to be involved in emotion regulation [28]. In a study by Chirico and colleagues [30], the emotion regulation strategery of *reappraisal* (i.e., changing one’s thinking about a situation to change its meaning) was positively and significantly predicted by positive emotions (including awe). On the other hand, positive emotions negatively predicted the emotion regulation strategy of *suppression* (i.e., moderating the outward signs of emotional responding).

Awe has also been related to spirituality in a variety of ways. Work by [31] found awe to increase agency detection (i.e., the sense that events are non-random and generated by intentional non-human agents) and other research has found awe to relate to feelings of spirituality [10, 32]. Furthermore, a awe was found to decrease participants’ perceptions in the power of science-rooted explanations for theistic concepts (such as God or religion [33]). Additionally, more recent work has found that engaging with science simultaneously promotes analytical thinking, as well as promotes belief in abstract representations of God through feelings of awe and self-transcendence [34].

Finally, there is a growing body of literature that has found awe experience to relate to prosocial values, motives, and behaviors [18, 35–37]. For example, awe elicited through recall and video tasks have been associated with prosociality [38]. Additionally, research found that awe was associated with increased ethical decision making [18] and other work found awe increased participants’ intentions to be generous and help others in need [39]. In fact, Rudd and colleagues [6] also found that awe was associated with an increased willingness to volunteer to help others and that this relationship was mediated by awe’s effect on time, with participants’ feeling as though they had more time to spare. Elicitors involving nature have also been shown to increase prosocial behavior, such as an increased willingness to donate [37] and other work found that awe elicitors (involving both nature and non-nature) weakened participants’ desire to obtain money [40]. Furthermore, awe elicited through recall and video tasks was shown to decrease aggression [38].

## What is awe-some?

A methodological challenge in studying awe involves finding a stimulus that will effectively and reliably elicit the emotion. Great variability in awe induction techniques exists, for instance, one situation asked participants to describe the feeling of awe to an alien with no knowledge of human emotion, while another situation involved exposing participants to a massive tyrannosaurus rex skeleton [41]. The most common elicitor of awe is nature, usually presented in image and videos [6, 7, 31, 32, 37]. One study even had participants stand in a grove of tall trees to elicit awe through nature in vivo [18]. Other studies have used images of tall buildings to elicit awe [17, 18]. Other common elicitors used in research involve exposing participants to music [7, 42], as well as, the use of recollection or writing tasks, where participants are prompted to recall or write about a time in their life when they had experienced awe [6, 41].

Researchers have been leveraging technological advances to elicit awe more effectively [43–45]. For instance, a recent study exposed participants (*N =* 16) to virtual environments and asked them to give ratings of their self-reported awe on a single-item measure designed for the study that ranged from 0–100 [44]. The mean score of self-reported awe was 79.7 and ratings of awe were significantly higher for participants who experienced goosebumps compared with those who did not (*t*(14) = 2.82, *p* < .001, *d* = 1.42). Another study used the AWE-S to evaluate the degree to which participants (*N =* 55) experienced awe during a virtual reality training designed to elicit awe, where participants were randomly assigned to awe-inspiring or neutral virtual environments [45]. Those exposed to awe-inspiring environments reported significantly higher scores of awe than the neutral group (*t*(52) = 2.095, *p* = .041, *d* = 1.08). Furthermore, the vastness and connectedness facets of awe were also significantly higher in the control group (vastness *t*(52) = 2.05,*p* = .036,*d* = 1.68); connectedness (*t*(52) = 2.12,*p* = .034,*d* = 1.64).

Another viable elicitor of awe is psychedelic experience. In fact, within the realm of psychedelic science, awe has been suggested as a potential mechanism of change in psychedelic experiences characterized by mystical-type qualities [46]. In a naturalistic online sample (*N =* 684) researchers recruited participants who both used psychedelic substances and those who did not, including a measure of “awe-proneness” or dispositional awe in the survey [11]. The results of this survey suggested that dispositional awe played a central role in the positive developmental outcomes associated with psychedelic use, finding that awe-proneness significantly predicted personality adjustment (β = 0.36, *p*< .001) and personality growth (β = 0.40, *p*< 0.001). Researchers also followed 30 participants who were randomly assigned to microdose psilocybin or placebo over the course of two three-week periods, with a three week break in-between [47]. Awe was measured using 5-items created specifically for the study, which were then summed into a total awe score. Participants reported more awe during the psilocybin microdosing condition compared to the placebo condition (*F*(1, 29) = 8.309,*p* = .007,η^2^ = .223). These more powerful, experiential elicitors of awe are promising avenues through which the field may study awe in real-time.

## Rationale

Since the development of the AWE-S [5], researchers have been better able to measure awe, as well as the facets associated with awe. Nevertheless, recent literature continues to include single-item assessments, or shorter adaptations created for the purposes of a given study. Especially in settings involving trying to capture awe in vivo, such as virtual reality and psychedelic settings, the 30-item original version may produce substantial participant burden and/or interfere with emotion manipulation. In this current study, we set out to replicate the validity of the original AWE-S using psychedelic samples, as the psychedelic state can be conceptually considered one of the most “intense” experiences of awe that can be elicited in vivo. We also derive a 12-item short-form of the AWE-S, which may be used by researchers to reduce both participant burden and to promote the use of an accessible, validated measure to enhance the rigor of awe research.

## Overview of the studies in the paper

In Study 1, we conduct an exploratory factor analysis and confirmatory factor analysis to replicate the factor structure of the AWE-S scale in a psychedelic sample. In Study 2, we utilize this sample to develop a short-form of the AWE-S, which is referred to as the AWE-SF. In Study 3, we replicate the confirmatory factor analysis from Study 2 in a larger and more diverse psychedelic sample. In Study 4, we used this sample to correlate the AWE-SF with relevant mental health outcomes. In Study 5, we further establish the validity of the AWE-SF by exploring correlations between the 30-item AWE-S and 12-item AWE-SF in a psychedelic sample with related measures of emotion (mDES), dispositional emotion (DPES), and personality variables used in the original AWE-S validation study. Finally in study 6 we extend our analysis to explore the predictive validity of the AWE-SF using the same emotion and personality measures, and other commonly used measures from psychedelic research (MEQ, CEQ) and well-being related outcomes (SWLS, PEQ-WB, Psychological Richness). Pre-registrations for Study 5 and 6 are available at https://doi.org/10.17605/OSF.IO/VY3QA. This manuscript’s minimal data set has been made publicly available via an Open Science Framework repository. It can be retrieved using the following URL: https://osf.io/xt4fa.

## Study 1—Exploratory factor analysis

In study 1, the full 30 items from the AWE-S were administered to 1,416 participants as part of a larger survey on psychedelic use among Canadian adults. Participants were asked to consider and rate awe experience in the context of their most positive psychedelic experience. Following data collection, we performed Exploratory Factor Analysis (EFA) on these items.

### Method

#### Participants

Data for this study were collected as part of a larger cross-sectional study entitled the *Canadian Psychedelics Survey (CPS)* [48], which looked at psychedelic use and self-reported outcomes among adults across Canada (*N* = 2,393). To participate in the study, respondents had to be 19 years or older, report past or current use of psychedelics, have the capacity to consent for themselves, and be able to read, write and speak English. Informed consent was gathered online, and all data was collected anonymously. However, participants could provide an email address to be entered into a draw to win one of 3 X $500 Amazon gift cards as compensation for taking the time to participate in the study, after which all email addresses gathered for the draw were immediately destroyed. The survey received ethics approval from Advarra (protocol # Pro00059863), and was co-sponsored by SABI Mind, the Multidisciplinary Association of Psychedelic Studies Public Benefits Corp. (MAPS PBC), and Psygen Industries. It was distributed January 14–28, 2022 via NGOs like MAPS Canada, the Psychedelic Association of Canada, and the Canadian Drug Policy Coalition, as well as social media. Respondents completed the survey online on REDCap, a HIPAA- and PIPEDA-compliant electronic data capture system. Online consent was sought and received from all participants.

To conduct exploratory factor analysis on the AWE-S scale, this study only included those participants who responded to the AWE-S, which was included in the larger CPS. Regarding awe in the CPS survey, participants were asked to complete the *Awe Experience Scale* (AWE-S) specifically as the items pertained to their psychedelic experience. Specifically, the survey asked:

*Have you ever had what you would describe as an* ***"intense"*** *but largely positive psychedelic experience?*

*Participant characteristics*. Of the total 2,393 people who participated in the CPS, 1,416 had experienced a largely positive psychedelic experience, and thus, responded to the AWE-S. This subset of the CPS sample (*n* = 1,416) was used to conduct EFA in this study.

This portion of the CPS sample was largely white (81.91%), 53.1% male, with a mean age of 38.57 (*SD =* 12.80). For full demographic information please see S1 Table.

#### Procedure

The full survey was administered across Canada to adults aged 19 years or older, with the aim of better understanding psychedelic use via self-description [48]. For the current study, only data for participants responding to the AWE-S were used in analyses. Participants were asked to first to endorse that they had had a profoundly positive psychedelic experience before gaining access to the AWE-S questions.

#### Measures

As part of the CPS, the *Awe Experience Scale* (AWE-S; [5, 26]) was administered. The scale consists of 30-items total, with five-items each for the six key domains of awe experience: altered time perception, self-diminishment, connectedness, vastness, physical sensations, and need for accommodation. The items are presented on a 7-point scale, from 1 (“strongly disagree”) to 7 (“strongly agree).

#### Data analyses

Study 1 focused on exploring the factor structure of the AWE-S in this sample using exploratory factor analysis (EFA). In the original AWE-S paper factor solutions were generated using SAS with Promax rotation, in this paper, SPSS with Promax rotation was used. We also report (within sample) CFA and inter-factor correlations using Confirmatory Factor Analysis (CFA). SPSS AMOS was used to compute the Comparative Fit Index (CFI), as well as the Root Mean-Square Error or Approximation (RMSEA) for both the AWE-S.

### Results

Our aim was to replicate the EFA conducted by Yaden and colleagues [5] in a psychedelic sample. Exploratory factor analysis (EFA) was conducted on all AWE-S items to examine how the top loading items in this sample compared with the Yaden and colleagues original paper. Replicating the 2019 paper, the EFA (with Promax rotation) produced a 6-factor solution with 5-itmes per factor. As in the 2019 paper, each of these factors had strong internal reliability: (F1) altered time perception α = .91; (F2) self-diminishment α = .91; (F3) connectedness α = .95; (F4) vastness (a) = .94; (F5) physical sensations α = .91; (F6) need for accommodation α = .91.

Factor loadings were adequate and comparable to those from the original study. Table 1 shows the factor loadings for our sample.

**Table 1:** Awe-S item loadings.

			Factors			
Item	1	2	3	4	5	6
I noticed time slowing.	0.85	-	-	-	-	-
I sensed things momentarily slow down.	0.83	-	-	-	-	-
I felt my sense of time change.	0.80	-	-	-	-	-
I experienced the passage of time differently.	0.78	-	-	-	-	-
I had the sense that moment lasted longer than usual.	0.77	-	-	-	-	-
I felt that my sense of self was diminished.	-	0.88	-	-	-	-
I experienced a reduced sense of self.	-	0.88	-	-	-	-
I felt my sense of self shrink.	-	0.85	-	-	-	-
I felt my sense of self become somehow smaller.	-	0.84	-	-	-	-
I felt small compared to everything else.	-	0.56	-	-	-	-
I experienced a sense of oneness with all things.	-	-	.85	-	-	-
I felt a sense of communion with all living things.	-	-	.83	-	-	-
I had the sense of being connected to everything.	-	-	.79	-	-	-
I had a sense of complete connectedness.	-	-	.79	-	-	-
I felt closely connected to humanity.	-	-	.78	-	-	-
I perceived something that was much larger than me.	-	-	-	.80	-	-
I felt the presence of greatness.	-	-	-	.80	-	-
I felt that I was in the presence of something grand.	-	-	-	.77	-	-
I experienced something greater than myself.	-	-	-	.75	-	-
I perceived vastness.	-	-	-	.62	-	-
I had goosebumps.	-	-	-	-	.84	-
I gasped.	-	-	-	-	.83	-
I had chills.	-	-	-	-	.82	-
I felt my jaw drop.	-	-	-	-	.77	-
I felt my eyes widen.	-	-	-	-	.76	-
I felt challenged to understand the experience.	-	-	-	-	-	.87
I found it hard to comprehend the experience in full.	-	-	-	-	-	.85
I struggled to take in all that I was experiencing at once.	-	-	-	-	-	.84
I felt challenged to mentally process what I was experiencing.	-	-	-	-	-	.75
I tried to understand the magnitude of what I was experiencing.	-	-	-	-	-	.67

n = 1416

While conducted in the same sample and therefore limited in value, we conducted confirmatory factor analysis (CFA). The full 30-item, six factor AWE-S in this study revealed an adequate confirmatory fit index (CFI; .930) and root mean square error approximation (RMSEA; .053), with 90% confidence intervals of .051 and .054 according to standard CFA benchmarks described by [49].

We calculated the inter-factor correlations for the AWE-S in our sample. All factors showed moderate to strong positive correlations with one another (see Table 2).

**Table 2:** Inter-factor correlations of the AWE-S factors.

	1.	2.	3.	4.	5.	6.
F1. Time	-					
F2. Self-loss	.48**	-				
F3. Connection	.46**	.42**	-			
F4. Vastness	.44**	.46**	.74**	-		
F5. Physical	.32**	.35**	.41**	.49**	-	
F6. Accommodation	.34**	.44**	.29**	.39**	.44**	-

*n =* 1416

** = p < .001

### Discussion

EFA on the AWE-S items in this sample replicated the original AWE-S, items loaded around six stable and reliable factors. Furthermore, like the original AWE-S, these factors demonstrated strong internal consistency.

## Study 2

Using this same sample from the CPS survey *(n* = 1,146) we explored the potential for the development of a short-form of the AWE-S. This was intended to develop a short-form version of the scale (12 questions) that could reduce participant burden.

### Method

#### Participants

Participants were the same as subset of the CPS sample from Study 1 (*n* = 1,416).

#### Procedure

The data were collected in the same way as in Study 1, where participants who endorsed having a profoundly positive psychedelic experience were then asked to fill out the AWE-S items.

#### Measures

The top two loading items from each factor of the original AWE-S from Study 1 were selected to explore a 12-item, six factor short form version of the AWE-S (referred to as the AWE-SF). Though these differ slightly from the top two loading items in the original AWE-S paper, the content of the items, as well as the factor loadings were extremely similar. Additionally given that this was a larger, updated, and psychedelic-specific sample (who may have experienced awe more intensely), we determined it would be appropriate to select the top 2 loading items from this study to compose the short-form measure (AWE-SF).

#### Data analyses

Study 2 focused on confirming the factor structure of the AWE-S short form (called AWE-SF hereafter) using Confirmatory Factor Analysis (CFA). SPSS AMOS was used to compute the Comparative Fit Index (CFI), as well as the Root Mean-Square Error or Approximation (RMSEA) for the AWE-SF.

### Results

#### Confirmatory factor analysis

CFA of items on the AWE-SF indicated excellent CFI (.989) and RMSEA (.034), with 90% confidence intervals of .028 and .040. These results are comparable to the AWE-S model fit (Study 1b).

#### Inter-factor correlations

We calculated the inter-factor correlations AWE-SF in our sample. Like the AWE-S, all factors showed moderate to strong positive correlations with one another (Table 3).

**Table 3:** Inter-factor correlations of the AWE-SF factors.

	1.	2.	3.	4.	5.	6.
F1. Time	-					
F2. Self-loss	.35**	-				
F3. Connection	.38**	.36**	-			
F4. Vastness	.34**	.36**	.68 **	-		
F5. Physical	.27**	.30**	.39 **	.43 **	-	
F6. Accommodation	.27**	.38**	.26 **	.31 **	.39**	-

*n =* 1416

** = p < .001

## Discussion Study 2

In this study, we developed a short-form, 12-item version of the Awe Experience Scale (AWE-S), which we call the Awe Experience Scale–Short Form (AWE-SF). Using the same subset of the CPS sample from Study 1 (*n* = 1,416), we selected the top two loading items from each factor of the original AWE-S to create the short-form. In Study 2, we confirm the factor structure of the AWE-SF, which demonstrate excellent fit indices which are comparable to the original scale. Additionally, we show that the AWE-S and AWE-SF show comparable strengths in correlations predicting aspects of psychedelic experience and mental health.

## Study 3

The factor structure of the short-form of the awe experience scale (AWE-SF) was confirmed in a *separate* Canadian Psychedelic Sample that was larger and more diverse than the sample used for study 1 and 2. Participants in this survey were part of the *Global Psychedelic Survey (GPS)* [50] were recruited online using social media and snowball sampling techniques from a larger geographic area, resulting in a larger and more diverse psychedelic sample.

### Method

#### Participants

The study received ethics approval from Advarra (protocol # Pro00071490), which oversaw Canadian subjects and determined that this international study was otherwise exempt from IRB oversight in other jurisdictions under Department of Health and Human Services regulation 45 CFR 46.104(d)(2). Online consent was sought and received from all participants. Of the N = 6,045 people who participated in the GPS [50], 4,783 participants reported that they had experienced a “Intense” psychedelic experience––which was not valenced positively or negatively, responded to the AWE-SF. The subset of this sample which completed the AWE-SF in full (*n* = 4,745) was used to conduct CFA in this study.

*Participant characteristics*. Of the total 6,045 people who participated in the GPS, 4,745 had experienced an intense psychedelic experience, and thus responded to the AWE-SF. This subset of the GPS sample (*n* = 4,745) was used to conduct CFA in this study. This portion of the GPS sample was largely Caucasian/European (76.71%), 47.50% female, with a mean age of 41.14 (*SD =* 13.78). For full demographic information please see S2 Table.

#### Procedure

The full survey was administered globally to adults aged 19 years or older, with the aim of better understanding psychedelic use via self-description. For the current study, only data for participants responding to the AWE-S were used in analyses. Participants were asked to first to endorse that they had had an intense psychedelic experience before gaining access to the AWE-S questions. Then participants were asked to consider awe experience *in the context of* their an “intense” psychedelic experience. Specifically, the survey asked:

*“Have you ever had what you would describe as an* ***INTENSE*** *experience while using psychedelics?”*

Participants who responded “Yes” would be directed to respond to the AWE-SF questions.

#### Measures

The two items for each subscale of the AWE-SF established in Study 2 were selected to confirm the structure of the AWE-SF in a new sample.

#### Data analyses

Planned analysis included Confirmatory Factor Analysis (CFA) to confirm the factor structure of the AWE-SF in a larger and more diverse sample. SPSS AMOS was used to compute the Comparative Fit Index (CFI), as well as the Root Mean-Square Error or Approximation (RMSEA) for both the AWE-S.

### Results

#### Confirmatory factor analysis

CFA of items on the AWE-SF in this larger sample indicated excellent CFI (.993) and RMSEA (.031), with 90% confidence intervals of .027 and .035.

#### Inter-factor correlations

We calculated the inter-factor correlations for the AWE-SF in this larger sample. All factors showed moderate to strong positive correlations with one another (Table 4).

**Table 4:** Inter-factor correlations of the AWE-SF factors.

	1.	2.	3.	4.	5.	6.
F1. Time	-					
F2. Self-loss	.29**	-				
F3. Connection	.28**	.26**	-			
F4. Vastness	.34**	.30**	.61 **	-		
F5. Physical	.23**	.21**	.30 **	.34 **	-	
F6. Accommodation	.13**	.31**	.37 *	.15 **	.22**	-

*n =* 4745

* = p < .05

** = p < .001

## Discussion Study 3

In this study, we confirmed the factor structure of the AWE-SF in a separate, larger, and more diverse sample (*n =* 4745).

## Study 4

The factor structure of the short-form of the AWE-S was confirmed in the Canadian Psychedelic Sample and we replicated this finding in a larger and more diverse psychedelic sample (GPS). In this larger sample used in study 4 (GPS), we also examine the relationship between scores on the AWE-SF to relevant outcomes related to well-being.

### Method

#### Participants

The same subset of participants from Study 3 who completed the AWE-SF in full (*n* = 4,745) was used in this additional analysis.

#### Procedure

Correlations were computed between the AWE-SF and measures related to mental health and well-being.

#### Measures

The AWE-SF, which measures a brief, acute mental state was correlated with various measures that reflect more trait-like measures of mental health and persisting positive or negative changes to well-being attributed to the psychedelic experience.

*Persisting positive or negative effects from experience on well-being*. The well-being item of the Persisting Effects Questionnaire (PEQ [51, 52]) was adapted for use in this study. Specifically, participants were asked to respond to the following question:

*Do you believe that your* ***MOST INTENSE EXPERIENCE*** *and your contemplation of that experience led to a* ***POSITIVE*** *or* ***NEGATIVE*** *change in your* ***CURRENT*** *sense of personal well-being or life satisfaction?*

Scores ranged from -3 to 3, with higher scores indicating a greater positive change in personal well-being or life satisfaction.

*Depressive symptoms—PHQ-9*. The Patient Health Questionnaire-9 (PHQ-9 [53]) is a 9-item self-report instrument used to assess depressive symptom severity "over the past 2 weeks". Participants respond to questions about the intensity of symptoms on a 4-point Likert-type scale ranging from 0 (“not at all”) to 3 (“nearly every day”). Unlike the PHQ-8, this measure includes a ninth item that assesses thoughts of self-harm and suicide. Scores are summed with higher scores indicating a greater severity of depressive symptoms.

*Anxiety—GAD-7*. The Generalized Anxiety Disorder-7 (GAD-7; [54]) questionnaire is a 7-item self-report instrument used to assess the severity of anxiety symptoms "over the last two weeks". Participants respond to questions about the intensity of symptoms on a 4-point Likert-type scale ranging from 0 (“not at all”) to 3 (“nearly every day”). Scores are summed with higher scores indicating a greater severity of depressive symptoms.

#### Data analyses

Correlation analyses were conducted to examine the relationship between the AWE-SF total scale and sub-scales with the well-being (adapted PEQ item), depression severity (PHQ-9) and anxiety severity (GAD-7).

### Results

The AWE-SF and each of the subscales were found to positively correlated with the adapted well-being outcome on the PEQ, as well as inversely correlated with measures of depression and anxiety (see Table 5).

**Table 5:** AWE-SF correlations with relevant outcomes.

	PEQ (*n =* 4740)	GAD-7 (*n* = 4645)	PHQ-9 (*n* = 4639)
Awe TOTAL	.377**	-.124**	-.179**
F1. Time	.161**	-.036*	-.071**
F2. Self-loss	.172**	-.104**	-.120**
F3. Connection	.448**	-.178**	-.222**
F4. Vastness	.472**	-.187**	-.237**
F5. Physiological	.181**	.004^ns^	-.039*
F6. Accommodation	.028 ^ns^	.011^ns^	-.007^ns^

*n =* 4745

* = p < .05

** = p < .001, ns = not significant

## Study 4 discussion

In this study, we began to establish the predictive validity of the AWE-SF in a psychedelic sample for outcomes related to mental health. AWE-SF and each of the facets were inversely correlated with measures of depression and anxiety. Additionally, total AWE-SF and facet scores were positively associated with a single-item well-being outcome measuring persisting positive or negative effects on well-being attributed to a psychedelic experience. This indicates that individuals who experienced high levels of awe during their most “intense” psychedelic experience also experienced greater degrees of well-being and lower severity of both depression and anxiety.

## Study 5

In Study 5, our aim was to compare the predictive validity of the original 30-item AWE-S to the 12-item AWE-SF on outcomes relevant to awe experience in a new sample (third described so far). The pre-registration for this study (and Study 6) is available at: https://doi.org/10.17605/OSF.IO/VY3QA. IRB approval was obtained for both Study 5 and 6 from Johns Hopkins University IRB (Protocol Number #IRB00298239). Written informed consent was collected online from study participants.

### Method

#### Participants

Participants for this survey were recruited using the *Prolific* survey recruitment platform, with the aim of recruiting a representative sample.

*Participant characteristics*. Participants in this sample (*n =* 476) were largely white (67.44%), 52.5% female, with a mean age of 28.87 (*SD* = 10.92). Please See, S3 Table for full demographic information.

#### Procedure

Participants were asked initial screening questions within the *Prolific* platform to determine eligibility. To be eligible for the study participants have been over the age of 18 and reported having had a psychedelic drug experience. The study was intentionally described broadly as “A study about memorable past experiences” to avoid solely recruiting individuals who may have been biased towards responding positively when asked about prior psychedelic drug experiences. If participants were eligible to participate, they were redirected to the informed consent and survey hosted in *Qualtrics*, an online survey platform. After providing their consent to participate, participants then responded to a recall prompt asking:

*Please take a moment to recall your* ***most memorable*** *psychedelic experience. This experience should be the most memorable time you felt the effects of a psychedelic.*

Participants were then asked to fill out several measures related to the experience they wrote about including questions about awe-experience, mystical experience, challenging experience, and persisting well-being effects attributed to the psychedelic experience (specific measures are described below), followed by more global measures of experience including questions about dispositional positive emotion, life satisfaction, psychological richness, and personality. The order in which these measures were presented in both blocks was randomized to minimize the influence of order effects. Finally, participants were asked to respond to demographic questions and questions about the context of their psychedelic drug experience.

#### Measures

*Awe*. The Awe Short-Form (AWE-SF) measure developed in Study 2 and used in Study 3–4 was also used in this study.

To replicate the outcomes associated with the original AWE-S [5], the following outcome measures were used in this study.

*Positive and negative emotions–State measure*: *Modified Differential Emotion Scale (mDES*; *[55]).* The mDES asks participants to rate their strongest experience of each of 20 specific emotions on a 5-point Likert scale (0 = Not At All to 4 = Extremely). This scale assesses experiences of discrete emotions, both positive and negative.

*Positive emotions–Trait measure: Dispositional Positive Emotion Scale (DPES; [56]).* The DPES is a 38-item self-report questionnaire that consists of 7 subscales (Joy, Contentment, Pride, Love, Compassion, Amusement, Awe) made up of 5–6 items each. Each scale is rated on a 7-point Likert scale (1 = Strongly disagree to 7 = Strongly agree). This scale measures one’s dispositional tendencies to feel positive emotions towards others in their daily lives. Items are averaged, yielding a range from 1 to 7. Higher scores indicate greater levels of positive emotion.

*Personality*. To account for individual differences in participants’ personality, participants completed the *Ten Item Personality Inventory (TIPI*; [57]), which assesses each of the Big-Five personality dimensions via two items for each dimension on a 7-point scale (1 = Disagree strongly to 7 = Agree strongly).

*Single item awe measure*. We also included a single-item awe measure which asked participants to describe whether their experience involved a “Sense of awe or awesomeness”, on a 6-point scale (0 = “None; not at all” to 5 = “Extreme (more than ever before in my life)”).

*Data analyses*. Correlations were conducted to determine the degree to which the original AWE-S and AWE-SF were associated with relevant emotion, well-being, and psychedelic outcomes.

### Results

#### Establishing initial predictive validity

Correlations were conducted to compare the association between the AWE-S and AWE-SF regarding outcomes relevant to awe experience such as positive and negative emotions, dispositional affect, and personality traits (Table 6).

**Table 6:** Correlations between AWE-S and AWE-SF on relevant outcomes.

Outcome Measures	AWE-S	AWE-SF
**Single Item Awe Measure**		
“Sense of awe or awesomeness”.	.395**	.407**
**mDES–Positive Emotions**		
Awe, Wonder, Astonishment	.301**	.307**
Inspired, Uplifted, Elevated	.349**	.356**
Serene, Content, Peaceful	.338**	.342**
Grateful, Appreciative, Thankful	.359**	.352**
Love, Closeness, Trust	.321**	.319**
Interested, Alert, Curious	.265**	.261**
Hopeful, Optimistic, Encouraged	.310**	.309**
Amused, Fun-loving, Silly	.197**	.207**
Proud, Confident, Self-assured	.259**	.256**
Glad, Happy, Joyful	.224**	.226**
**mDES–Negative Emotions**		
Stressed, Nervous, Overwhelmed	.120*	.101*
Scared, Fearful, Afraid	.119*	.098*
Angry, Irritated, Annoyed	.114*	.103*
Disgust, Distaste, Revulsion	.088^ns^	.080^ns^
Sad, Downhearted, Unhappy	.162**	.144*
Ashamed, Humiliated, Disgraced	.121*	.104*
Embarrassed, Self-Conscious, Blushing	.174**	.160**
Hate, Distrust, Suspicious	.127*	.109*
Contemptuous, Scornful, Disdainful	.116*	.106*
Guilty, Repentant, Blameworthy	.167**	.160**
**DPES Items**		
Awe	.281**	.263**
Joy	.184**	.169**
Content	.160**	.151**
Pride	.175**	.165**
Love	.138*	.119*
Compassion	.189**	.168**
Humor	.140*	.132*
**TIPI**		
Extraversion	.042^ns^	.049^ns^
Agreeableness	.079^ns^	.071^ns^
Conscientiousness	-.010^ns^	.002^ns^
Emotional Stability	.050^ns^	.063^ns^
Openness to Experience	.135*	.124*

*N* = 476

* = p < .05

** = p < .001, ns = not significant

## Study 5 discussion

Correlations demonstrate that the AWE-S and the AWE-SF perform comparably when predicting outcomes related to awe experience such as positive and negative emotions, dispositional affect, and personality. The AWE-S and AWS-SF showed the strongest (moderate to strong) positive correlations with the single-item measure of awe. Notably, when awe belonged to a cluster “Awe, wonder, astonishment” in the mDES, other emotion clusters were more strongly associated with AWE-S and AWE-F, indicating both versions of the scale did not demonstrate strong discriminant validity among the positive emotion clusters.

## Study 6

In Study 6, we sought to replicate and extend the findings around initial and predictive validity from study 5, using the AWE-SF in a separate, larger sample (4th sample described so far) and evaluating both the total AWE-SF and factor level AWE-SF scores.

### Method

#### Participants

Participants for this survey were recruited using the *Prolific* survey recruitment platform, with the aim of recruiting a representative sample.

#### Participant characteristics

Participants in this sample (*n =* 1007) were largely white (70.31%), 52.93% female, with a mean age of 46.23 (*SD* = 63.21). Please See S4 Table for full demographic information.

#### Procedure

The same procedure used in Study 5 was followed for this study.

#### Measures

The same measures used in Study 5 were used in this study in addition to outcomes relevant to psychedelic experience and positive or negative persisting effects on well-being attributable to the experience.

We included the following measures of well-being, which has often been an outcome associated with awe experience.

*Life satisfaction*. The *Satisfaction with Life Sale (SWLS*; [58]) was used to assess life satisfaction. The SWLS is a short 5-item instrument designed to measure global cognitive judgments of satisfaction with one’s life.

*Psychological richness*. The *Psychologically Rich Life Questionnaire* [59] was included. This 17-item scale assessing a variety of interesting and perspective-changing experiences that make life ‘psychologically rich.’ Higher scores on this scale indicate a more psychologically rich life. Respondents use the following 7-point scale (1 = strongly to 7 = strongly agree).

Finally, we included the following outcome measures relevant to psychedelic experience.

*Mystical experience*. The *Mystical Experience Questionnaire (MEQ*; [60]) measured the presence of subjectively mystical-type experiences with 30 items on a 6-point scale (0 = “None; not at all” to 5 = “Extreme (more than ever before in my life)”).

*Challenging experience*. *The Challenging Experience Questionnaire (CEQ*; [61]) asks respondents to indicate the extent to which they experienced different phenomena during their experience (e.g., sadness, grief, fear, isolation, sense of dying) via 26 items on a 6-point scale (0 = None; not at all, 5 = Extreme).

*Persisting effects on well-being*. Three single-items items from the *Persisting Effects Questionnaire (PEQ;* Griffiths et al., 2011) were included. The PEQ is a non-validated measure of enduring effects attributed to psychedelic experiences [52] that has been used in several studies of psychedelics’ effects (e.g., [51, 62–64]). The PEQ questions included in this study asked participants to indicate the extent to which their psychedelic experience changed their personal well-being, increased a sense of life’s preciousness, and decreased death apprehension. Because each of these items addressed different persisting effects (with some variations in response options) and they were taken from different subsections of the PEQ, each one was considered a separate outcome measure.

#### Data analyses

Correlations were conducted to determine the degree to which the AWE-SF was associated with relevant emotion, well-being, and psychedelic outcomes. Linear regression analyses, controlling for age, gender, education, and socioeconomic status, were also conducted to better understand predictors of awe experience, as well as the degree to which awe experience predicted relevant psychedelic and well-being outcomes.

### Results

#### Replicating initial validity

We compared the AWE-SF with two other scales that measure emotions, including awe, the mDES (modified to be a state measure) and the D-PES (a trait measure). We also look at personality factors which have been demonstrated in past research to be related to awe. These measures were used to establish the initial validity in the original AWE-S study.

*State-level emotion–mDES*. The total AWE-SF score was positively and significantly correlated with every positive emotion cluster of the mDES, and most strongly correlated with the *Awe*, *Wonder and Astonishment cluster* (Table 7). This finding contradicts what was seen in Study 5, though this sample is double the size compared with the sample size of Study 5.

**Table 7:** Correlations of AWE-SF total and factors with mDES positive emotions.

Factors	AWE-SF Total	Time	Self-Loss	Connection	Vastness	Physical	Accommodation
Awe, Wonder, Astonishment	.40**	.15**	-.004	.59**	.62**	.23**	-.06
Inspired, Uplifted, Elevated	.35**	.10**	-.02	.59**	.58**	.21**	-.14**
Serene, Content, Peaceful	.32**	.09**	.01	.56**	.50**	.19**	-.15**
Grateful, Appreciative, Thankful	.37**	.09**	.05	.56**	.53**	.27**	-.10**
Love, Closeness, Trust	.34**	.11**	.02	.56**	.50**	.23**	-.12**
Interested, Alert, Curious	.35**	.14**	.04	.47**	.47**	.21**	.01
Hopeful, Optimistic, Encouraged	.36**	.11**	.02	.59**	.54**	.25**	-.13**
Amused, Fun-loving, Silly	.21**	.07*	-.08*	.39**	.35**	.17**	-.12**
Proud, Confident, Self-assured	.30**	.07*	-.002	.51**	.44**	.24**	-.14**
Glad, Happy, Joyful	.26**	.08*	-.08*	.52**	.47**	.17**	-.19**

n = 1007

** = p < .001

* = p < .05

Broadly, AWE-SF scores were most strongly and positively associated with the domains of connection and vastness, small to moderately positively associated with the physical domain, and were negatively correlated with accommodation and self-loss (though correlations were not significant for self-loss; see Table 6). This trend replicates the original results of the AWE-S using a non-psychedelic sample (see Yaden et al [5], Table 7).

We conducted a linear regression analysis of the positive mDES items to predict total AWE-SF score, while controlling for age, gender, education, and SES, and including each of the positive emotion mDES items as covariates. Multicollinearity diagnostics indicated no issues, as all Variance Inflation Factors (VIF) were below the commonly accepted threshold of 5. The independent predictors of the AWE-SF, which replicated the results of the AWE-S paper [5], were the following clusters: “awe, wonder, astonishment” (β = .245, *p* < .001), “grateful, appreciative, thankful” (β = .127, *p* = .009), “love, closeness, trust” (β = .102, *p* = .026), “interested, curious, alert” (β = .150, *p =* < .001). Like the original study, we found “glad, happy, joyful” to predict lower awe scores (β = -.235, *p* < .001). Unlike the original AWE-S, in our psychedelic sample, the cluster “hopeful, optimistic, encouraged” also predicted awe (β = .110, p = .036). Additionally, unlike the full AWE-S, in our sample “serene, content, peaceful” was not significantly associated with awe scores in either direction. In addition to the main predictors, the control variable gender also predicted awe (β = -.074, *p* < .008).

In a stark departure from the original AWE-S, in our psychedelic sample, the AWE-SF total score was significantly correlated with every negative emotion cluster (see Table 8).

**Table 8:** Correlations of AWE-SF total and factors with mDES negative emotions.

Factors	AWE-SF Total	Time	Self-Loss	Connection	Vastness	Physical	Accommodation
Stressed, Nervous, Overwhelmed	.11**	.12**	.32**	-.29**	-.20**	.15**	.38**
Scared, Fearful, Afraid	.09*	.10**	.30**	-.31**	-.22**	.13**	.37**
Angry, Irritated, Annoyed	.08*	.05	.28**	-.19**	-.15**	.16**	.20**
Disgust, Distaste, Revulsion	.11**	.07*	.28**	-.18**	-.14**	.19**	.21**
Sad, Downhearted, Unhappy	.11**	.06	.34**	-.19**	-.14**	.13**	.27**
Ashamed, Humiliated, Disgraced	.14**	.09*	.34**	-.17**	-.14**	.19**	.25**
Embarrassed, Self-Conscious, Blushing	.14**	.07*	.28**	-.12**	-.10**	.19**	.24**
Hate, Distrust, Suspicious	.11**	.10*	.28**	-.18**	-.17**	.18**	.24**
Contemptuous, Scornful, Disdainful	.15**	.09*	.29**	-.11**	-.08*	.22**	.20**
Guilty, Repentant, Blameworthy	.16**	.07*	.32**	-.13**	-.08**	.19**	.25**

n = 1007

** = p < .001

* = p < .05

In a reversal of the trend seen in the positive emotion cluster, self-loss and accommodation were moderately positively associated with every negative emotion cluster, while connection and vastness were moderately negatively associated with these clusters. The physical domain remained positively associated with the negative emotion clusters, as it did with the positive emotion clusters.

We also conducted a linear regression analysis of the negative mDES items to predict total AWE-SF score, while controlling for age, gender, education, and SES, and including each of the negative emotion mDES items as covariates. Multicollinearity diagnostics indicated no issues, as all Variance Inflation Factors (VIF) were below the commonly accepted threshold of 5. The only independent predictor of total awe score was the negative emotion cluster “contemptuous, scornful, disdainful” (β = .143, *p* = .009). Unlike the original AWE-S paper, we did not find “stressed, nervous, overwhelmed” to be predictive of total awe score in either direction. In addition to the main predictors, the covariate gender also predicted awe (β = -.065, *p* = .037).

*Trait-level emotion—D-PES*. Like the original AWE-S study, we also included a trait measure of emotion, which measures one’s dispositional tendency to experience awe. We found that the AWE-SF was significantly and positively associated with all the other positive emotions. Like the original AWE-S paper, the correlation between total AWE-SF and dispositional awe was stronger than for any other positive emotions. Additional relationships between AWE-SF domains and D-PES items are available in Table 9.

**Table 9:** Correlations of AWE-SF total and factors with D-PES.

Factors	AWE-SF Total	Time	Self-Loss	Connection	Vastness	Physical	Accommodation
Awe	.29**	.11**	.13**	.30**	.30**	.20**	.08**
Joy	.21**	.04	.06	.27**	.24**	.18**	.02
Content	.15**	.02	.01	.25**	.20**	.10*	-.03
Pride	.15**	.06	-.01	.23**	.20**	.10*	-.01
Love	.17**	.03	.07*	.21**	.15**	.16**	.03
Compassion	.20**	.08*	.12**	.18**	.18**	.12**	.10*
Humor	.15**	.07*	.05	.12**	.14**	.17**	.04

n = 1007

** = p < .001

* = p < .05

We conducted a linear regression analysis of the DPES items to predict total AWE-SF score, while controlling for age, gender, education, and SES, and including each of the DPES items as covariates. Multicollinearity diagnostics indicated no issues, as all Variance Inflation Factors (VIF) were below the commonly accepted threshold of 5. The only independent predictor of total AWE-SF scores was dispositional awe, (β = 0.282, *p* < .001). In addition to the main predictors, the control variable gender also predicted awe (β = -.076 *p* = .013).

*Big five personality*. Like the original AWE-S paper, we examined the relationship between AWE-SF scores and Big-5 personality factors as measured by the TIPI. In our psychedelic sample, Openness to Experience was the only factor to be significantly correlated with awe (see Table 10). Openness to Experience was also the only significant predictor of total awe scores (β = .150, *p <* .001), after controlling for age, gender, education, and SES and including the TIPI items as covariates. Multicollinearity diagnostics indicated no issues, as all Variance Inflation Factors (VIF) were below the commonly accepted threshold of 5. In addition to the main predictors, the control variable gender also predicted awe (β = -.071, *p* = .024).

**Table 10:** Correlations of the AWE-SF total and factors with TIPI.

Factors	AWE-SF Total	Time	Self-Loss	Connection	Vastness	Physical	Accommodation
Extraversion	.05	-.01	-.05	.13**	.12**	.07*	-.06
Agreeableness	.06	.002	-.01	.15**	.12**	.02	-.06
Conscientiousness	-.03	.003	-.05	.04	.01	-.05	-.06
Emotional Stability	-.01	-.03	-.07*	.09*	.08*	-.02	-.11**
Openness to Experience	.15**	.06	.03	.19**	.23**	.07*	-.02

n = 1007

** = p < .001

* = p < .05

Overall, initial validity of the AWE-SF aligns with the initial validity established in the original AWE-S paper. Like the AWE-S, the AWE-SF was largely predictive of the same trait and state positive emotions. Unlike the AWE-S, the AWE-SF as measured in our psychedelic sample, was also significantly associated with negative emotions. Factors such as Connection and Vastness tended to be associated with positive emotional experiences, Self-Loss and Accommodation tended to be associated with negative emotional experiences, and the Physical domain of awe experience was associated with both positive and negative trait and state measures of emotion. Like the original AWE-S, Openness to Experience was predictive of higher total awe scores. Additionally, self-loss and accommodation were significantly negatively associated with Emotional Stability (which is the inverse of Neuroticism [65]).

#### AWE-SF and psychedelic-relevant outcomes

To extend the analyses beyond initial validation, we also included psychedelic relevant outcome measures to establish the predictive validity of the AWE-SF in these samples.

*Mystical experience–MEQ*. Total AWE-SF scores were strongly and significantly associated with overall mystical experience scores, as well as each of the factors of the Mystical Experience Questionnaire (See Table 11). Furthermore, we conducted a linear regression to determine if AWE-SF facets predicted mystical experience, with covariates of age, gender, education, and SES and the facets of awe as covariates. Multicollinearity diagnostics indicated no issues, as all Variance Inflation Factors (VIF) were below the commonly accepted threshold of 5. The only factors of awe which were independent predictors of MEQ-Total scores were Connection (β = .387, *p* < .001), Vastness (β = .402, *p* < .001), and Time (β = .110, *p* < .001). None of the control variables also predicted mystical experience.

**Table 11:** Correlations of AWE-SF total and factors with Mystical Experience (MEQ-30).

Factors	AWE-SF Total	Time	Self-Loss	Connection	Vastness	Physical	Accommodation
**MEQ Total Score**	.65**	.34**	.22**	.73**	.74**	.37**	.12**
Mystical Experience	.63**	.27**	.19**	.77**	.75**	.35**	.06
Positive Mood	.42**	.16**	-.02	.65**	.64**	.24**	-.11**
Transcendence	.56**	.46**	.37**	.30**	.38**	.32**	.37**
Ineffability	.49**	.33**	.20**	.37**	.45**	.25**	.30**

n = 1007

** = p < .001

* = p < .05

*Challenging experience–CEQ*. Total AWE-SF scores were also strongly and significantly associated with each of the dimensions of challenging experience (see Table 12). The overall trend reflected in the correlations indicates that time, self-loss, and accommodation are significantly positively associated with each domain of challenging experience, while connection and vastness are significantly negatively correlated with each domain of challenging experience.

**Table 12:** Correlations of AWE-SF total & factors with Challenging Experience (CEQ-30).

Factors	AWE-SF Total	Time	Self-Loss	Connection	Vastness	Physical	Accommodation
**CEQ Total Score**	.19**	.18**	.39**	-.25**	-.17**	.21**	.40**
Fear	.10**	.13**	.33**	-.33**	-.24**	.15**	.40**
Grief	.17**	.12**	.38**	-.19**	-.12*	.18**	.33**
Physical Distress	.26**	.21**	.32**	-.10**	-.05	.32**	.34**
Insanity	.18**	.21**	.35**	-.24**	-.16**	.15**	.42**
Isolation	.12**	.14**	.35**	-.28**	-.18**	.11**	.34**
Death	.18**	.15**	.32**	-.12**	-.06	.16**	.25**
Paranoia	.13**	.09*	.31**	-.15**	-.13**	.17*	.21**

n = 1007

** = p < .001

* = p < .05

This trend persists when examining whether facets of awe predict challenging experience, controlling for age, gender, education, and SES and facets of AWE-SF as covariates. The AWE-SF factors of self-loss (β = .291, *p <* .001), accommodation (β = .198, *p* < .001), physical (β = .173, *p* < .001), and time (β = .092, *p* = .001) were predictive of overall challenging experience scores in the positive direction while connection (β = -.280, *p* < .001) and vastness (β = -.136, *p* = .001) were predictive of overall challenging experience in the negative direction. Multicollinearity diagnostics indicated no issues, as all Variance Inflation Factors (VIF) were below the commonly accepted threshold of 5. None of the covariates predicted challenging experience.

In terms of psychedelic related outcomes, the AWE-SF significantly predicts both mystical and, to a lesser extent, challenging experiences, though different facets of awe seem to be related to these different variables related to psychedelic experiences. Mystical-type experience is largely predicted by the AWE-SF facets of connection, vastness, and time. On the other hand, challenging experiences are largely predicted by self-loss, accommodation, time, and physical facets of the AWE-SF. Furthermore, lower scores of connections and vastness on the AWE-SF predict stronger endorsement of the various domains of challenging experience.

*AWE-SF and well-being outcomes*. Finally, as awe experiences as measured by the original AWE-S have been linked to well-being outcomes, we included well-being related outcomes measures to explore the predictive validity of the AWE-SF in our psychedelic sample.

*Life satisfaction*. The AWE-SF total score was positively and significantly associated with SWLS scores (see Table 13) and the only AWE-SF factors to be significantly positively associated with SWLS scores were the facets connection, vastness, and physical. We conducted a linear regression examining whether facets of AWE-SF predict life satisfaction, controlling for age, gender, education and SES, and the facets of awe as covariates. Multicollinearity diagnostics indicated no issues, as all Variance Inflation Factors (VIF) were below the commonly accepted threshold of 5. The only facets of the AWE-SF which were independent predictor of life satisfaction was connection (β = .228, *p <* .001) in the positive direction, and time (β = -.063, *p* = .042) in the negative direction. In addition to the main predictors, the control variables SES (β = .375, *p* < .001) and education (β = .099, *p* = .001) also predicted life satisfaction scores.

**Table 13:** Correlations of the AWE-SF total and well-being measures.

	AWE-SF Total	Time	Self-Loss	Connection	Vastness	Physical	Accommodation
**Life Satisfaction Total Score (SWLS)**	.13**	.004	.02	.22**	.15**	.09*	-.02
**Psychologically Rich Life**	.28**	.15**	.12**	.24**	.25**	.21**	.11**

n = 1007

** = p < .001

* = p < .05

#### Psychological richness

The AWE-SF total score positively and significantly associated with Psychologically Rich Life Scores (see Table 13). The AWE-SF facets of connection (β = .114, *p* = .018), vastness (β = .100, *p =* .040), and physical (β = .088, *p* = .014) were the only independent predictors of Psychological Richness, when controlling for age, gender, education, and SES, and the psychological richness items as covariates. Multicollinearity diagnostics indicated no issues, as all Variance Inflation Factors (VIF) were below the commonly accepted threshold of 5. In addition to the main predictors, the control variables SES (β = .112, *p* < .001) and education (β = .068, *p* = .038) also predicted life satisfactions cores.

*Persisting well-being effects*. While life satisfaction and psychological richness ask participants about their global sense of well-being more broadly in a trait-like way which limits inferences, we also included items from the persisting effects questionnaire to examine aspects positive or negative effects on well-being attributed to the psychedelic experience participants were asked to reflect on. Total AWE-SF scores were significantly correlated with each of the PEQ well-being items in the direction indicating the experience had a more positive than negative effect on well-being (see Table 14).

**Table 14:** Correlations of the AWE-SF total and persisting-effects items.

	AWE-SF Total	Time	Self-Loss	Connection	Vastness	Physical	Accommodation
***PEQ–Single Items***							
Well-Being & Life Satisfaction Item	.34**	.14**	.12**	.40**	.42**	.18**	.05
Preciousness of Life Item	.44**	.17**	.22**	.42**	.44**	.30**	.14**
Reduced Death Apprehension Item	.38**	.15**	.25**	.33**	.35**	.27**	.08*

n = 1007

** = p < .001

* = p < .05

When controlling for age, gender, education, and SES, and including each of the AWE-SF subscales as covariates, several facets of the AWE-SF predicted PEQ single-item outcomes. Multicollinearity diagnostics indicated no issues, as all Variance Inflation Factors (VIF) were below the commonly accepted threshold of 5 for each of the PEQ outcomes. Connection (β = .196, *p* < .001) and Vastness (β = .265, *p* < .001) were the only independent predictors of PEQ Well-Being & Life Satisfaction. None of the control variables were significant predictors for this item.

Connection (β = .202, *p* = < .001), Vastness (β = .233, *p =* < .001), Self-Loss (β = .090, *p =* .005), and Physical (β = .077, *p =* .020) were also the only independent predictors of PEQ Preciousness of Life. In addition to the main predictors, the covariate SES (β = .075, *p* = .013) was also a predictor of PEQ Preciousness of Life.

Lastly, Vastness (β = .167 *p* = < .001), and Self-Loss (β = .179, *p* = < .001), Connection (β = .138, *p* = .003), and Physical (β = .094, *p* = .007) were the only independent predictors of PEQ item Reduced Death Apprehension. None of the control variables also predicted this item.

## Study 6 discussion

In Study 6, we provide evidence to support the initial validity of the AWE-SF by replicating and extending the findings from Study 5. Like the original 30-item version [5], the AWE-SF shows strong positive correlations with positive emotion states, as well as strong associations with trait level positive emotions, particularly dispositional awe. Also like the original AWE-S, the AWE-SF was significantly correlated with openness to experience, which has been associated with awe across the literature [7, 66, 67]. Study 6 also establishes the predictive validity of the AWE-SF for psychedelic samples, where awe experience is associated with psychedelic and well-being outcomes, highlighting the importance of various facets of awe differentially. Vastness and Connection were associated with positive emotional states, mystical experience, and well-being, as well as persisting effects such as the sense that life is precious and general well-being and life satisfaction as a result of psychedelic experience. On the other hand, self-loss and accommodation were associated with challenging experience and negative emotion states. We discuss the implications of these findings in the next section.

## Overall discussion

We sought to develop and validate a short-form of the Awe Experience Scale (AWE-SF) using large samples of participants reflecting on their psychedelic experiences. This work extends and replicates the original conceptual framework of awe involving vastness and accommodation outlined by Keltner and Haidt [1], as well as the work by Yaden and colleagues [5] who included the facets of connection, vastness, self-loss, time, and physiological changes. By testing the AWE-S (Study 1) and the AWE-SF (Study 2–6) in psychedelic samples, we were able to measure the facets of awe as they relate to one particular trigger of intense awe experiences. Across the 6 studies, we offer strong and compelling evidence that the AWE-SF, like the original 30-item version, is a valid and reliable tool for measuring awe experience. Additionally, we provide evidence for the predictive validity of the AWE-SF in predicting outcomes related to psychedelic experience and well-being more broadly.

### Initial validity and factor structure

The results of Studies 1–3 consistently and reliably replicated the six-factor structure of the original AWE-S, where vastness, accommodation, self-loss, connection, time perception, and physical sensations were related yet distinct factors. Furthermore, in Study 2 and 3, the 12-item AWE-SF items demonstrated excellent fit indices that are comparable to the original 30-item version. Study 5 provided additional evidence of the AWE-SF’s validity by demonstrating strong associations with relevant emotional and psychological outcomes. Consistent with past research [5, 22, 56], the AWE-SF was positively associated with state and trait positive emotions. Additionally, the AWE-SF was significantly associated with openness to experience, a personality trait which has been linked to awe in previous studies [7, 66, 67]. Lastly, Study 5 and 6 demonstrated that awe experience is associated with both positive and negative emotional states, which aligns with past conceptualizations that awe as a *complex* emotion [2] that may also involve fear, a sense of being overwhelmed, and threat-based appraisals [3, 4]. These findings suggest that not only is the AWE-SF congruent with the larger 30-item version, but that it is also associated with aspects found to be associated with awe experience, and often measured using techniques other than the AWE-S.

Our findings from Studies 5 and 6 extend what has been studied regarding facets of awe experience and emotions for more intense elicitors of awe. For instance, Study 5 demonstrated that the facets of connection and vastness were more strongly associated with and predicted by positive emotional states. While the facets of self-loss and accommodation were more strongly associated with and predicted by negative emotional states. This trend reproduces what was seen in recent work by Pizzolante and colleagues [45] who used the AWE-S to measure awe elicited by virtual reality settings (described in the introduction). In their work, they demonstrated not only those experiences with awe-inspiring virtual environments led to higher ratings of awe when compared to a control group, but that connection and vastness were the facets of awe that were rated significantly higher in the group exposed to awe-inspiring virtual environments. Given that virtual reality is another powerful and reliable elicitor of awe experience, like psychedelic experiences, it may be the case that as the field continues to study awe in using more intense elicitors, we may be better able to understand the role of the facets of awe in these contexts.

### Psychedelic and well-being outcomes

Throughout studies 1–6 we used samples that asked participants to reflect on psychedelic experiences, and in Study 6 we included outcomes measures relevant to psychedelic science [52] including the *Mystical Experience Questionnaire* [60], the *Challenging Experience Questionnaire* [61], and the *Persisting Effects Questionnaire* [51]. We found a similar trend to that observed with emotional states, where the facets of connection and vastness were more likely to be associated with and predictive of Mystical experiences and the PEQ single items assessing for well-being and a sense of life’s preciousness, while Self-Loss and Accommodation were more likely to be associated with Challenging experiences and reduced death apprehension (PEQ single item). Awe has been conceptualized to be a potential mechanism of change in psychedelic science [46] and we believe that our findings begin to add evidence to such conceptualizations.

Mystical experience as measured by the MEQ is characterized by the following four factors: 1) *Mystical*, or the subjective experience of both internal and external unity, sacredness, and noetic quality; 2) *Positive Mood*, involving several positive emotions; 3) *Transcendence of time or space*, involving changes to the passage of time and one’s surroundings; and, 4) *Ineffability*, or the sense that the experience is beyond words [60]. The facets of the AWE-SF that were most strongly associated with and predictive of mystical experience were connection and vastness. Connection as measured by the AWE-SF taps into a “connectedness” and “communion” with “everything” and “all living things”, which speaks to the self-transcendent quality of this facet. Vastness as measures by the AWE-SF also involves self-transcendence, along with an encounter with “something grand” or “something greater than myself”. This suggests that intense awe experiences that are characterized by connection and vastness and mystical experiences may involve similar psychological processes.

Similarly, the specific Self-Loss and Accommodation facets of the AWE-SF are most strongly associated with and predictive of Challenging Experiences. Psychedelic Science acknowledges that psychedelic experience even in clinical trial settings is not without risks [68]. The CEQ measures difficult and/or challenging experiences that have been reported in psychedelic experience and has 7 factors: *Fear*, *Grief*, *Physical Distress*, *Insanity*, *Isolation*, *Death*, and *Paranoia* [61]. Self-Loss as measured by the AWE-SF measures the degree to which the self is “diminished” or “shrink[s]” and the Accommodation facet asks whether participants feel “challenged to mentally process” their experience and whether the experience was “hard to comprehend”. The Self-loss and Accommodation facets of awe reflect the fearful and disorienting nature of challenging experiences, where individuals become less secure in their sense of self and their understanding of their experience.

That intense psychedelic experiences are associated with different facets of awe, also reflects the different sorts of outcomes associated with mystical and challenging experience. Mystical experiences tend to be associated with well-being [69], while challenging experiences tend to be associated (in some cases) with emotional breakthrough experiences [70–72]. In line with this trend psychedelic experiences characterized by connection and vastness in our sample were predictive of well-being and the sense that life is precious (as measured by the PEQ), while the psychedelic experiences characterized by self-loss and accommodation were predictive of reduced apprehension of death (as measured by the PEQ). Therefore, it may be the case that intense awe experiences may be better characterized and understood through the facets of awe with which they are associated rather than an overall awe score, which may obscure and fail to capture these differences. By measuring which facets of awe individuals are experiences in psychedelic clinical trials, for example, guides may better determine whether to structure integration around enhancing well-being (mystical experiences characterized by connection and vastness) or to facilitate processing an emotional breakthrough (challenging experiences characterized by self-loss and accommodation).

Finally, in terms of well-being, the same facets of awe (connection and vastness) which predicted mystical experience and positive emotional states, also were associated with life satisfaction and the sense that one’s life is psychologically rich. What this may signal is the importance of self-transcendent experiences of “connection” or “communion” as well as the sense that one is perceiving a world that involves vast stimuli that are “greater than themselves”. These findings are in line with the already extensive literature demonstrating that awe (regardless of how it is measured) is associated with well-being [6, 27, 73]. Future research might explore the causal relationships between intense experiences of awe, the specific facets that are associated with these experiences, and the well-being outcomes they are connected to.

### Limitations

There are several limitations of this study worth noting. First, the exclusive use of psychedelic experience recall in each of our samples may limit the generalizability of our findings to other contexts. This is especially true when it comes to the more nuanced findings pertaining to the facets of awe, which seem to operate differently for different kinds of acute subjective psychedelic experiences. Relatedly, while we asked participants to reflect on past psychedelic experiences to capture a strong elicitor of awe, we did not directly measure awe during the course of a psychedelic experience (or in the context of a clinical trial). On the other hand, many studies have examined awe outside of the psychedelic domain and this is among the first few to investigate awe specifically in the domain of psychedelic experiences. Lastly, while we did recruit large, representative (Study 5–6) and global (Study 3–4) samples, there may be limitations on the degree to which cultural influences on awe experience and measurement may be captured given the heterogeneity of the samples.

In the future, researchers might explore the validity of the AWE-SF in homogenous cross-cultural settings where self-transcendent experience may be feature largely in daily life. Recent work by Stellar and colleagues [74] found that fear was more likely to be involved in awe experiences in a Chinese sample versus a US sample, where awe experiences were characterized by positive emotions. Incorporating the AWE-SF into these sorts of investigations would allow for a more precise understanding of which specific facets of awe might be driving these differences. This sort of work would also help to refine the external validity of the measure. Future research might also attempt to evaluate awe experience close in time to the eliciting event, for example shortly following a psychedelic experience in the context of a clinical trial, to determine whether scores on the facets of awe that are recalled are like scores on facets of awe in vivo. Finally, there is potential for psychedelic clinical trials to incorporate the AWE-SF and consider the information about which facets are endorsed by participants to guide research questions.

## Conclusion

Since the initial conceptualization of awe in the psychological research literature [1] there has been a growing interest in studying awe experience in a variety of contexts, using various elicitors, and associating the experience with various outcomes (often associated with well-being). However, there has notably been an under-emphasis on valid and reliable measurement. Many studies have utilized single-item measures, others have created scales for the use in single-research studies. The only valid and reliable measure of awe is the Awe Experience Scale (AWE-S), though it is lengthy at 30-items. The development of a short form is meant to serve two distinct purposes, first, to reduce participant burden by reducing the number of items from 30 to 12 and second, to encourage the use of valid and reliable measures of awe to strengthen the rigor of the field and allow for meaningful comparisons between studies and across varying elicitors of awe. The Awe Experience Scale-Short Form was created for these purposes (and is available in S2 File).

## Supporting information

S1 Table Demographic information for Study 1–2 (n = 1416). (DOCX)

S2 Table Demographic information for Study 3–4 (n = 4745). (DOCX)

S3 Table Demographic information for Study 5 (N = 476). (DOCX)

S4 Table Demographic information for Study 6 (N = 1007). (DOCX)

S1 File Inclusivity in global research. (DOCX)

S2 File Awe experience scale–Short form. (DOCX)
