# Supplementary material for: Developing a short form of the Awe Experience Scale (AWE-SF) in psychedelic samples
Source: PLoS One. 2024 Dec 4;19(12):e0314469. doi: 10.1371/journal.pone.0314469 (PMC11616893; doi:10.1371/journal.pone.0314469)
Supplement: S1 Table — (DOCX) [file pone.0314469.s001.docx]

**Supplemental Table 1**

*Demographic Information for Study 1-2 (n*=1416*)*

| **Age** |  |  |  |
| --- | --- | --- | --- |
|  | Mean | 38.57 |  |
|  | SD | 12.80 |  |
|  |  | **N** | **% Sample** |
| **Race** |  |  |  |
|  | White | 1232 | 81.91 |
|  | Black or African American | 19 | 1.26 |
|  | Asian | 64 | 4.26 |
|  | South Asian | 33 | 2.19 |
|  | Aboriginal or First Nation | 50 | 3.32 |
|  | Metis | 50 | 3.32 |
|  | Other | 56 | 3.72 |
| **Ethnicity** |  |  |  |
|  | Hispanic | 35 | 2.50 |
|  | Non-Hispanic | 1381 | 97.50 |
| **Gender** |  |  |  |
|  | Male | 752 | 53.10 |
|  | Female | 585 | 41.30 |
|  | Non-binary | 62 | 4.40 |
|  | Prefer not to self-describe | 12 | 0.80 |
|  | Prefer not to say | 5 | 0.40 |
| **Self-reported Socioeconomic Status** | | |  |
|  | Less than $10,000 | 272 | 19.20 |
|  | $10,000-$39,999 | 212 | 15.00 |
|  | $40,000-$69,999 | 131 | 9.30 |
|  | $70,000-$99,999 | 310 | 21.90 |
|  | $100,000-$129,999 | 269 | 19.00 |
|  | $130,000-$159,999 | 41 | 2.90 |
|  | Over $160,000 | 181 | 12.80 |
| **Education** |  |  |  |
|  | No high school degree/GED equivalent | 28 | 2.00 |
|  | High school/GED | 214 | 15.10 |
|  | Technical and non-university degree | 361 | 25.50 |
|  | University Degree | 492 | 25.50 |
|  | Graduate degree | 236 | 16.70 |
|  | Doctorate or Professional Degree | 85 | 6.00 |
| **Marital Status** | |  |  |
|  | Married | 393 | 27.80 |
|  | Widowed | 19 | 1.30 |
|  | Divorced | 83 | 5.90 |
|  | Separated | 56 | 4.00 |
|  | In a Domestic Partnership or Civil Union | 323 | 22.80 |
|  | Single, but cohabiting with a significant other | 137 | 9.70 |
|  | Single, never married | 405 | 98.70 |
| **Canadian Province or Territory** | | | |
|  | Alberta | 570 | 40.30 |
|  | British Columbia | 337 | 23.80 |
|  | Manitoba | 27 | 1.90 |
|  | New Brunswick | 12 | 0.80 |
|  | Newfoundland | 5 | 0.40 |
|  | Northwest Territories | 1 | 0.10 |
|  | Nova Scotia | 32 | 2.30 |
|  | Nunavut (Territory) | 2 | 0.10 |
|  | Ontario | 322 | 22.70 |
|  | Prince Edward Island | 2 | 0.10 |
|  | Quebec | 88 | 6.20 |
|  | Saskatchewan | 12 | 0.80 |
|  | Yukon (Territory) | 6 | 0.40 |
