# Supplementary material for: Developing a short form of the Awe Experience Scale (AWE-SF) in psychedelic samples
Source: PLoS One. 2024 Dec 4;19(12):e0314469. doi: 10.1371/journal.pone.0314469 (PMC11616893; doi:10.1371/journal.pone.0314469)
Supplement: S2 Table — (DOCX) [file pone.0314469.s002.docx]

**Supplemental Table 2**

*Demographic Information for Study 3-4 (n=*4745*)*

| **Age** |  |  |  |
| --- | --- | --- | --- |
|  | Mean | 43.14 |  |
|  | SD | 13.78 |  |
|  |  | **N** | **% Sample** |
| **Race & Ethnicity** | | | |
|  | African | 9 | 0.19 |
|  | Black/African diaspora | 60 | 1.26 |
|  | Caucasian/European | 3640 | 76.71 |
|  | East Asian | 41 | 0.86 |
|  | Indigenous | 32 | 0.67 |
|  | Latin, Hispanic, Central and South American | 230 | 4.85 |
|  | Oceanian | 99 | 2.09 |
|  | South Asian | 58 | 1.22 |
|  | South East Asian | 28 | 0.59 |
|  | West Central Asian, Middle Eastern and North African | 40 | 0.84 |
|  | Prefer not to say | 31 | 0.65 |
|  | Other | 62 | 1.31 |
|  | Multiracial | 415 | 8.75 |
| **Gender** |  |  |  |
|  | Male | 2255 | 47.50 |
|  | Female | 2328 | 49.10 |
|  | Non-binary | 113 | 2.40 |
|  | Prefer not to self-describe | 49 | 1.0 |
| **Self-reported Socioeconomic Status** | | |  |
|  | Very low income/well below average | 248 | 5.20 |
|  | Low income/below average | 761 | 16.00 |
|  | Middle income/about average | 2090 | 44.00 |
|  | High income/above average | 1356 | 28.60 |
|  | Very high income/well above average | 290 | 6.10 |
| **Education** |  |  |  |
|  | No high school degree/GED equivalent | 70 | 1.50 |
|  | High school/GED | 599 | 12.60 |
|  | Technical and non-university degree | 624 | 13.20 |
|  | University Degree | 1600 | 33.70 |
|  | Graduate degree | 1276 | 26.90 |
|  | Doctorate or Professional Degree | 576 | 12.10 |
| **Marital Status** | |  |  |
|  | Single, never married | 1162 | 24.50 |
|  | Single, but cohabiting with a significant other | 608 | 12.80 |
|  | In a Domestic Partnership or Civil Union | 472 | 9.90 |
|  | Married | 1701 | 35.80 |
|  | Divorced/Separated | 711 | 15.00 |
|  | Widowed | 91 | 1.90 |
| **Country of Residence** | | | |
|  | American Samoa | 1 | 0.00 |
|  | Andorra | 1 | 0.00 |
|  | Angola | 2 | 0.00 |
|  | Antarctica | 1 | 0.00 |
|  | Argentina | 13 | 0.30 |
|  | Australia | 602 | 12.70 |
|  | Austria | 9 | 0.20 |
|  | Belgium | 7 | 0.10 |
|  | Bosnia and Herzegovina | 1 | 0.00 |
|  | Brazil | 35 | 0.70 |
|  | Bulgaria | 1 | 0.00 |
|  | Canada | 453 | 9.50 |
|  | Chile | 11 | 0.20 |
|  | Colombia | 9 | 0.20 |
|  | Costa Rica | 8 | 0.20 |
|  | Croatia | 4 | 0.10 |
|  | Cyprus | 2 | 0.20 |
|  | Czechia | 5 | 0.10 |
|  | Denmark | 11 | 0.20 |
|  | Ecuador | 3 | 0.10 |
|  | Estonia | 6 | 0.10 |
|  | Eswatini | 1 | 0.00 |
|  | Finland | 5 | 0.10 |
|  | France | 12 | 0.30 |
|  | Georgia | 1 | 0.00 |
|  | Germany | 57 | 1.20 |
|  | Greece | 8 | 0.20 |
|  | Guatemala | 2 | 0.00 |
|  | Hong Kong | 5 | 0.10 |
|  | Hungary | 12 | 0.30 |
|  | Iceland | 1 | 0.00 |
|  | India | 15 | 0.30 |
|  | Indonesia | 3 | 0.10 |
|  | Ireland | 16 | 0.30 |
|  | Israel | 22 | 0.50 |
|  | Italy | 19 | 0.40 |
|  | Japan | 2 | 0.00 |
|  | Kenya | 2 | 0.00 |
|  | Latvia | 2 | 0.00 |
|  | Luxembourg | 1 | 0.00 |
|  | Malaysia | 1 | 0.00 |
|  | Malta | 1 | 0.00 |
|  | Mexico | 43 | 0.90 |
|  | Nepal | 1 | 0.00 |
|  | Netherlands | 53 | 1.1 |
|  | New Zealand | 42 | 0.90 |
|  | North Macedonia | 1 | 0.00 |
|  | Norway | 26 | 0.50 |
|  | Paraguay | 2 | 0.00 |
|  | Peru | 3 | 0.10 |
|  | Philippines | 4 | 0.10 |
|  | Poland | 61 | 1.30 |
|  | Portugal | 19 | 0.40 |
|  | Puerto Rico | 1 | 0.00 |
|  | Romania | 7 | 0.10 |
|  | Russian Federation | 4 | 0.10 |
|  | Serbia | 3 | 0.10 |
|  | Singapore | 1 | 0.00 |
|  | Slovakia | 4 | 0.10 |
|  | South Africa | 16 | 0.30 |
|  | Spain | 36 | 0.80 |
|  | Sri Lanka | 3 | 0.10 |
|  | Sweden | 32 | 0.70 |
|  | Switzerland | 17 | 0.40 |
|  | Thailand | 9 | 0.20 |
|  | Turkey | 2 | 0.00 |
|  | Turks and Caicos | 1 | 0.00 |
|  | Ukraine | 6 | 0.10 |
|  | United Arab Emirates | 1 | 0.00 |
|  | UK and Northern Ireland | 144 | 3.00 |
|  | United States of America | 2812 | 59.30 |
|  | Uruguay | 3 | 0.10 |
|  | Vietnam | 3 | 0.10 |
