# Supplementary material for: Developing a short form of the Awe Experience Scale (AWE-SF) in psychedelic samples
Source: PLoS One. 2024 Dec 4;19(12):e0314469. doi: 10.1371/journal.pone.0314469 (PMC11616893; doi:10.1371/journal.pone.0314469)
Supplement: S3 Table — (DOCX) [file pone.0314469.s003.docx]

**Supplemental Table 3**

*Demographic Information for Study 5 (N=476*)

| **Age** |  |  |  |
| --- | --- | --- | --- |
|  | Mean | 28.87 |  |
|  | SD | 10.92 |  |
|  |  | **N** | **% Sample** |
| **Race** |  |  |  |
|  | White | 321 | 67.44 |
|  | Black or African American | 67 | 14.08 |
|  | Asian | 28 | 5.88 |
|  | Native Hawaiian or Other Pacific Islander | 1 | 0.21 |
|  | Multiracial | 47 | 9.87 |
|  | Other | 13 | 2.73 |
| **Ethnicity** |  |  |  |
|  | Hispanic | 422 | 88.7 |
|  | Non-Hispanic | 54 | 11.3 |
| **Sex** |  |  |  |
|  | Male | 226 | 47.5 |
|  | Female | 250 | 52.5 |
| **Gender** |  |  |  |
|  | Male | 222 | 46.6 |
|  | Female | 234 | 49.4 |
|  | Non-binary | 13 | 2.7 |
|  | Genderqueer or genderfluid | 3 | 0.6 |
|  | Agender | 1 | 0.2 |
|  | Other | 2 | 0.4 |
| **Self-reported Socioeconomic Status** | | |  |
|  | Poor | 41 | 8.6 |
|  | Working class | 179 | 37.6 |
|  | Middle class | 180 | 37.8 |
|  | Upper-middle class | 69 | 14.5 |
|  | Upper class | 7 | 1.5 |
| **Education** |  |  |  |
|  | No high school degree/GED equivalent | 4 | 0.8 |
|  | High school/GED | 146 | 30.7 |
|  | Associate or Arts (AA) | 87 | 18.3 |
|  | Bachelor's Degree (graduate college) | 160 | 33.6 |
|  | Master's degree | 70 | 14.7 |
|  | MD/PhD/JD | 9 | 1.9 |
| **Marital Status** | |  |  |
|  | Married or living with partner | 232 | 48.7 |
|  | Divorced/separated | 64 | 13.4 |
|  | Widowed | 14 | 2.9 |
|  | Never married | 166 | 34.9 |
| **Self-reported Religiosity** | |  |  |
|  | Not religious | 211 | 44.3 |
|  | Slightly religious | 118 | 24.8 |
|  | Moderately religious | 99 | 20.8 |
|  | Very religious | 48 | 10.1 |
| **Self-reported Spirituality** | |  |  |
|  | Not spiritual | 79 | 16.6 |
|  | Slightly spiritual | 120 | 25.2 |
|  | Moderately spiritual | 144 | 30.3 |
|  | Very spiritual | 133 | 27.9 |
