# Supplementary material for: Developing a short form of the Awe Experience Scale (AWE-SF) in psychedelic samples
Source: PLoS One. 2024 Dec 4;19(12):e0314469. doi: 10.1371/journal.pone.0314469 (PMC11616893; doi:10.1371/journal.pone.0314469)
Supplement: S4 Table — (DOCX) [file pone.0314469.s004.docx]

**Supplemental Table 4**

*Demographic Information for Study 6 (N=*1007)

| **Age** |  |  |  |
| --- | --- | --- | --- |
|  | Mean | 46.23 |  |
|  | SD | 63.21 |  |
|  |  | **N** | **% Sample** |
| **Race** |  |  |  |
|  | White | 708 | 70.31 |
|  | Black or African American | 129 | 12.81 |
|  | Asian | 47 | 4.67 |
|  | Native Hawaiian or Other Pacific Islander | 2 | 0.2 |
|  | American Indian or Alaska Native | 13 | 1.29 |
|  | Multiracial | 71 | 7.06 |
|  | Other | 37 | 3.67 |
| **Ethnicity** |  |  |  |
|  | Hispanic | 100 | 9.93 |
|  | Non-Hispanic | 907 | 90.07 |
| **Sex** |  |  |  |
|  | Male | 474 | 47.07 |
|  | Female | 533 | 52.93 |
| **Gender** |  |  |  |
|  | Male | 472 | 46.87 |
|  | Female | 507 | 50.35 |
|  | Transgender | 4 | 0.4 |
|  | Non-binary | 18 | 1.79 |
|  | Genderqueer or genderfluid | 2 | 0.2 |
|  | Agender | 2 | 0.2 |
|  | Unsure | 1 | 0.1 |
|  | Other | 1 | 0.1 |
| **Self-reported Socioeconomic Status** | | |  |
|  | Poor | 69 | 6.85 |
|  | Working class | 366 | 36.35 |
|  | Middle class | 429 | 42.6 |
|  | Upper-middle class | 134 | 13.31 |
|  | Upper class | 9 | 0.89 |
| **Education** |  |  |  |
|  | No high school degree/GED equivalent | 9 | 0.89 |
|  | High school/GED | 332 | 32.97 |
|  | Associate or Arts (AA) | 148 | 14.7 |
|  | Bachelor's Degree (graduate college) | 357 | 35.45 |
|  | Master's degree | 133 | 13.21 |
|  | MD/PhD/JD | 28 | 2.78 |
| **Marital Status** | |  |  |
|  | Married or living with partner | 511 | 50.74 |
|  | Divorced/separated | 120 | 11.92 |
|  | Widowed | 31 | 3.08 |
|  | Never married | 345 | 34.26 |
| **Self-reported Religiosity** | |  |  |
|  | Not religious | 492 | 48.86 |
|  | Slightly religious | 223 | 22.14 |
|  | Moderately religious | 199 | 19.76 |
|  | Very religious | 93 | 9.24 |
| **Self-reported Spirituality** | |  |  |
|  | Not spiritual | 191 | 18.97 |
|  | Slightly spiritual | 256 | 25.42 |
|  | Moderately spiritual | 325 | 32.27 |
|  | Very spiritual | 235 | 23.34 |
