# Supplementary material for: Developing a short form of the Awe Experience Scale (AWE-SF) in psychedelic samples
Source: PLoS One. 2024 Dec 4;19(12):e0314469. doi: 10.1371/journal.pone.0314469 (PMC11616893; doi:10.1371/journal.pone.0314469)
Supplement: S2 File — (DOCX) [file pone.0314469.s006.docx]

**Supplement 2**

**Awe Experience Scale – Short Form**

Administer the items below using the following scale points:

1 = "Strongly Disagree"

2 = "Moderately Disagree"

3 = "Somewhat Disagree

4 = "Neutral"

5 = "Somewhat Agree"

6 = "Moderately Agree"

7 = "Strongly Agree"

**Factor: Time**

Item 1: "I sensed things momentarily slow down."

Item 2: "I noticed time slowing.

**Factor: Self-Loss**

Item 1: "I felt my sense of self was diminished."

Item 2: "I felt my sense of self shrink."

**Factor: Connectedness**

Item 1: "I had the sense of being connected to everything."

Item 2: "I felt a sense of communion with all living things."

**Factor: Vastness**

Item 1: "I felt that I was in the presence of something grand."

Item 2: "I experienced something greater than myself."

**Factor: Physiological Changes**

Item 1: "I felt my jaw drop."

Item 2: "I had goosebumps."

**Factor: Accommodation**

Item 1: "I felt challenged to mentally process what I was experiencing."

Item 2: "I found it hard to comprehend the experience in full."

**Scoring:** Scores may be calculated by averaging the responses to a given factor and/or all items can be summed and then averaged as a “total awe” score.
